# Efficient Production of Broad-Spectrum Antimicrobials by *Paenibacillus polymyxa* OSY–EC Using Acid Whey-Based Medium and Novel Antimicrobial Concentration Approach

**DOI:** 10.3389/fbioe.2022.869778

**Published:** 2022-05-13

**Authors:** Ahmed G. Abdelhamid, Emily P. Campbell, Zach Hawkins, Ahmed E. Yousef

**Affiliations:** ^1^ Department of Food Science and Technology, The Ohio State University, Columbus, OH, United States; ^2^ Botany and Microbiology Department, Faculty of Science, Benha University, Benha, Egypt; ^3^ Department of Microbiology, The Ohio State University, Columbus, OH, United States

**Keywords:** acid whey, antimicrobial peptides, microbial bioreaction, food preservation, whey valorization, *Paenibacillus polymyxa*

## Abstract

Production of some antimicrobial peptides by bacterial producers is a resource-intensive process, thus, using inexpensive growth media and simplifying antimicrobial extraction and down-stream processing are highly desirable. Acid whey, a dairy industry waste, is explored as a medium for production of broad–spectrum antimicrobials from selected bacteriocinogenic bacteria. Neutralized and yeast extract-supplemented acid whey was suitable for production of antimicrobials by four tested strains, but *Paenibacillus polymyxa* OSY–EC was the most prolific antimicrobial producer. Concentrating synthesized antimicrobials during culture incubation using beads of polymeric adsorbent resin, followed by solvent extraction and freeze-drying, resulted in antimicrobials-rich powder (AMRP). Under these conditions, *P. polymyxa* OSY–EC produced paenibacillin, polymyxin E, and fusaricidin, which are active against Gram-positive and Gram-negative bacteria and fungi, respectively. When media containing 2x and 4x minimum inhibitory concentrations of AMRP were inoculated with *Listeria innocua* and *Escherichia coli*, microbial populations decreased by ≥4–log CFU ml^−1^ in tryptic soy broth and ≥3.5–log CFU ml^−1^ in milk. The antimicrobial mechanism of action of AMRP solutions was attributed to the disruption of cytoplasmic membrane of indicator strains, *L. innocua* and *E. coli*. These findings exemplify promising strategies for valorization of acid whey *via* microbial bioreactions to yield potent antimicrobials.

## 1 Introduction

Acid whey is a waste stream produced during the manufacture of certain cheeses and Greek yogurt, in which milk casein is coagulated by the action of lactic starter cultures (Chwialkowska et al., 2019). On the contrary, “sweet whey” is produced during manufacture of cheeses in which rennet is used to coagulate milk casein (Durham and Hourigan, 2007). Unlike acid whey, sweet whey is utilized efficiently by many cheese producers. During manufacture of cheese, it is estimated that 10 kg of milk results in 9 kg of cheese whey (Prazeres et al., 2012). Cheese whey has a high biological oxygen demand (27–60 g L^−1^) and chemical oxygen demand (50–70 g L^−1^), which make the treatment of dairy processing waste waters an expensive operation (Carvalho et al., 2013). Remarkably, just 50% of the cheese whey is transformed into valuable industrial products (Mollea et al., 2013; Chwialkowska et al., 2019).

Acid and sweet whey are different appreciably in composition and value. Acid whey (pH < 5) has higher content of lactic acid, phosphates, and total solids but lower protein and lactose levels than sweet whey (Jelen, 2011). The chemical composition of acid whey varies by the source of milk (e.g., sheep or cow), animal feed, health, and the stage of lactation (Chwialkowska et al., 2019). Disposal of acid whey poses a significant difficulty for the dairy industry, but two approaches for its utilization have been introduced lately (O’Leary et al., 1977; Chwialkowska et al., 2019); (i) production of whey powder, and (ii) microbial transformation into value–added products. The latter process exploits the presence of nutrients in acid whey to support microbial growth. Acid whey, for example, has been used to produce caproic acid and bioethanol. Currently, there is no record in published literature of the utilization of acid whey as a medium for production of antimicrobial peptides by microbial producers.

In the era of emerging antibiotic resistance (CDC, 2019), discovery of new antimicrobials is gaining priority in many research laboratories but streamlining methods of extraction of the newly discovered antimicrobials from complex culture media is equally important. The current study was initiated to utilize acid whey as a low–cost medium for production of beneficial antimicrobial peptides, and to simplify the methods used in their production and extraction. Utilization of acid whey in this manner could facilitate industrial production of these antimicrobial peptides, which are often produced in small quantities and nutrient–rich microbiological media are required for their production. Hence, the objectives of the current study were to (i) exploit acid whey as antimicrobial peptide production medium, using the recently discovered producer strain, *Paenibacillus polymyxa* OSY–EC (Campbell et al., 2021), (ii) develop a protocol for easy recovery of the antimicrobials from the production medium, and (iii) investigate potential applications and mode of action of extracted antimicrobials.

## 2 Materials and Methods

### 2.1 Bacterial Strains

Four bacteriocinogenic bacterial strains, *P. polymyxa* OSY–EC, *Bacillus velezensis* GF610, *B. velezensis* CE2, and *B. velezensis* OSY–S3 (Table 1) were screened in the current work for ability to produce antimicrobials in acid whey. These strains were transferred from their stock cultures into tryptic soy broth (TSB; Becton, Dickinson and Company, Franklin Lakes, NJ, United States) and incubated for 24 h at 37°C before use in various experiments. A panel of bacterial and fungal strains were tested for sensitivity to the antimicrobials produced by a test strain. The targeted bacterial strains were *Staphylococcus aureus* ATCC 6538, *Listeria innocua* ATCC 33090, *Pseudomonas aeruginosa* ATCC 9027, *Escherichia coli* ATCC 8739, *E. coli* k12, *Klebsiella pneumonia* 556, *Enterobacter gergoviae* 565, *Burkholderia cepacia* ATCC 25416, and *Serratia marcescens* 562. The targeted fungal strains were *Candia albicans* ATCC 10231 and *Aspergillus brasiliensis* ATCC 16404. The minimum inhibitory concentration (MIC) of the antimicrobials against the targeted strains was determined using the standard CLSI broth microdilution method (Clinical and Laboratory Standards Institute, 2015), as described later. Before use in the MIC experiment, the targeted bacterial strains were grown in TSB for 24 h at 37°C whereas *C. albicans* and *A. brasiliensis* were grown in TSB for 2 and 7 days, respectively, at 30°C. *L. innocua* ATCC 33090, *E. coli* k12, and *C. albicans* P57072 were used as indicator strains for anti–Gram positive, anti–Gram negative, and anti–fungal activity (He et al., 2007; Gerst et al., 2015; Hirakawa et al., 2015), respectively. All strains were obtained from the food microbiology laboratory collection, Department of Food Science and Technology, The Ohio State University, Columbus, Ohio, United States.

**TABLE 1:** The antimicrobial producer strains that were screened for antimicrobial production in modified cheese acid whey.

Bacterial strains	Antimicrobial characteristics (and antimicrobials produced)	References
*Paenibacillus polymyxa* OSY–EC	- Activity against Gram-positive bacteria (paenibacillin)—Activity against Gram-negative bacteria (polymyxin)—Antifungal (fusaricidin)	He et al. (2007); He et al. (2008); Huang and Yousef (2012); Campbell et al. (2021)
*Bacillus velezensis* GF610	- Activity against Gram-positive bacteria (Amyloliquecidin GF610)—Antifungal (difficidin, surfactin and fengycin)	Gerst and Yousef (2018); Gerst et al. (2021)
*Bacillus velezensis* CE2	Activity against Gram-positive bacteria (a lantibiotic)	Campbell et al. (2019)
*Bacillus velezensis* OSY–S3	Activity against Gram-positive bacteria, and antifungal agents (up to 12 antimicrobial agents encoded on the genome)	Gerst (2017); Gerst and Yousef (2018)

### 2.2 Cheese Acid Whey

Acid whey was procured from Superior Dairy Inc. (Canton, OH, United States) as a by–product from the production of cottage and ricotta cheeses. The composition of the acid whey was determined as follows. Lactose content was determined by an enzymatic “low-lactose determination” method (Mangan et al., 2019) using a commercial kit (Megazyme, Wicklow, Ireland). The protein content was determined by a dye-binding method (AOAC 967.12–1970, Protein in milk. Dye binding method I) using a protein analyzer (Sprint; CEM Corporation, Matthews, NC, United States). The ash content was determined (AOAC 945.46–1945, Ash of milk. Gravimetric method) using a microwave muffle furnace (CEM Corporation). Calcium concentration was determined by complexometric titration (Kamal, 1960). Fat content was determined as described by Cartwright et al., 2005, using a fat analyzer (SMART Trac II Analyzer; CEM Corporation). Measurement and adjustment of pH were done using pH meter (Mettler-Toledo Co., Columbus, OH, United States) equipped with a glass electrode, and calibrated at pH values of 4.0, 7.0 and 10.0, at 25°C.

### 2.3 Initial Screening of Antimicrobial Producers Using Acid Whey

Prior to use, acid whey was clarified by centrifugation to remove sediments and heated at 65°C for 30 min to decontaminate the product; no microorganisms were recoverable on microbiological media after heating. The acid whey was modified as follows: (a) pH adjustment to 6 or 7 with 5 N NaOH (Fisher Scientific, Fair Lawn, NJ, United States), (b) addition of ultra-high temperature (UHT) skim milk (1% w/v), and (c) supplementation with yeast extract (YE; Becton, Dickinson and Company) at 1% w/v level. Yeast extract was dissolved in water and autoclaved prior to addition to the whey.

The four selected antimicrobial producers were cultured at 1% (v/v) in 20 ml of the modified acid whey as shown in Table 2. The incubation was completed in 125–ml baffled Erlenmeyer flasks agitated at 150 revolutions/min (RPM) in a shaking incubator (New Brunswick Scientific, Edison, NJ, United States) at 30°C for 24 or 48 h. After incubation, cells of the producer bacteria were removed by centrifugation at 7,000 × g and 4°C for 20 min and the supernatant was collected and stored at 4°C until being used for antimicrobial testing, using the antimicrobial bioassay technique described later. Based on the antimicrobial activity assessment (Table 2), the highest antimicrobial producer in modified acid whey, under given bioreaction conditions, was selected for scaling up the antimicrobial production.

**TABLE 2:** Antimicrobial activity^a^ produced by strains of *Bacillus velezensis* (CE2, S3, GF610) and *Paenibacillus polymyxa* (OSY-EC) in acid whey (20 ml), which has been modified and pH-adjusted, after incubation at 30°C for 24 or 48 h in baffled Erlenmeyer flask with agitation at 150 RPM.

Bioreaction conditions			Activity against *Listeria innocua* ATCC 33090				Activity against *Escherichia coli* K12			
Acid whey supplementation	pH adjustment	Incubation time (h)	CE2	S3	GF610	OSY-EC	CE2	S3	GF610	OSY-EC
None	None (4.6)	24	—	—	—	—	—	—	—	—
None	6	24	++	—	+	—	—	—	—	—
1% Skim milk	6	24	—	+	—	—	—	—	—	+
1% Yeast extract	6	24	++	+	+	+	—	—	—	—
1% Yeast extract	7	24	+	+	+	++	—	+	—	+++
1% Yeast extract	7	48	+	+	++	+++	—	—	—	+++

a Degree of activity was determined based on observed diameter of inhibition area as follows: (—) not detected; (+) 5–8 mm; (++) 9–12 mm; (+++) 13 mm at least.

### 2.4 Scaled-Up Production and Concentration of Antimicrobials in Acid Whey

**FIGURE 1:**
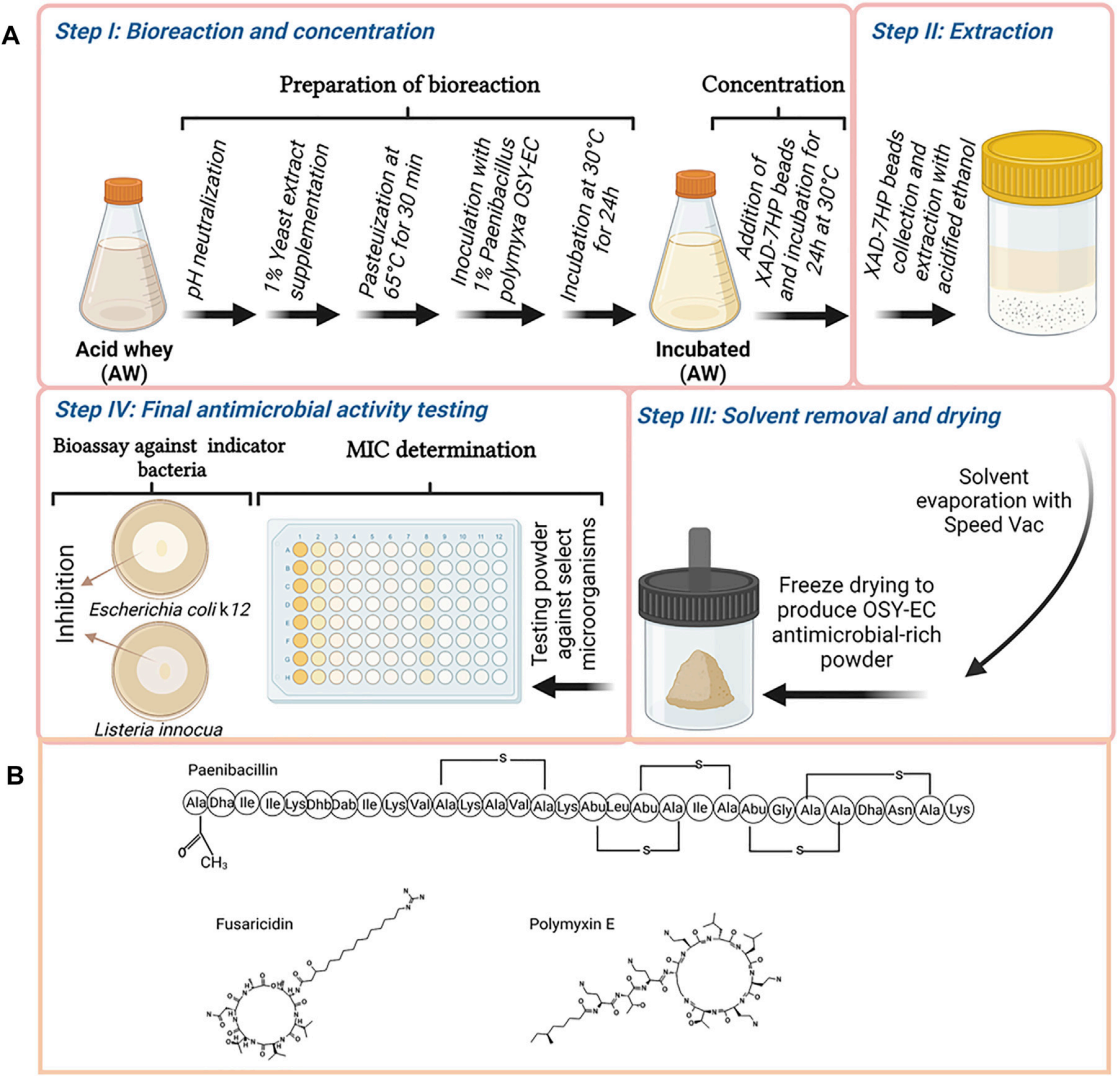
Graphical illustration of the procedure used in this study to produce antimicrobials by *Paenibacillus polymyxa* OSY–EC in acid whey-based medium, concentration of the produced antimicrobials using XAD-7HP polymeric resign, preparation of OSY–EC antimicrobials-rich powder, and testing product’s antimicrobial potency using antimicrobial bioassay or determination of the minimum inhibitory concentration **(A)**, and the structure of the produced compounds are shown **(B)**. Figure was created using Biorender.com.

According to the screening results (Table 2), *P. polymyxa* OSY–EC was selected for scaled–up production. The *P. polymyxa* OSY–EC strain is a mutant of the parent *P. polymyxa* OSY–DF (Campbell et al., 2021) from which paenibacillin and polymyxin were isolated previously (He et al., 2007) and a fusaricidin gene cluster was also detected (Huang and Yousef, 2012). A process, for simultaneous microbial growth and concentration of the produced antimicrobials, was developed as illustrated in Figure 1. Briefly, acid whey medium was ultrafiltered using 800 kDa membrane (Membrane Specialists, Hamilton, OH, United States) to remove sediments. The acid whey permeate (AWP) was modified by neutralization to pH 7.0 with 5 N NaOH, and supplementation with yeast extract (1%; wt/v). The modified acid whey was then pasteurized at 65°C for 30 min. The pasteurized acid whey–based medium was poured into four sterile 2-L flasks with a final content of 800 ml per flask. This was followed by inoculating *P. polymyxa* OSY–EC culture, at 1% (v/v) level, and incubating at 30°C for 48 h with shaking at 120 rpm. After the first 24 h of incubation, beads of a polymeric adsorbent resin (Amberlite XAD7HP; Sigma Aldrich, St. Louis, MO, United States), pasteurized at 65°C for 30 min, were added at 10% (wt/v) to the cultured acid whey to allow binding of antimicrobials with the resin. After the conclusion of incubation (a total of 48 h), the beads of the polymeric resin in each flask were collected and washed with 1 L of sterile deionized water, twice; This was followed by washing once with 500 ml of 30% ethanol. To elute the bound antimicrobials, the washed resin was resuspended in 250 ml of acidified 70% ethanol (pH 2.5) and the resin suspension was incubated at 22–25°C for 4 h with shaking at 130 rpm. After incubation, the acidified solvent containing the antimicrobials was collected, and then the antimicrobials were concentrated by evaporating the solvent (ethanol) using a vacuum concentrator (Automatic Environmental SpeedVac^®^ System, Savant, Hyannis, MA, United States) to yield semi–purified antimicrobial extract. The latter was dried using a freezer dryer (Labconco, Kansas, MO, United States) and the resulting freeze–dried antimicrobials-rich powder (designated as OSY–EC AMRP) was used throughout the study. After suspension in water, the MIC of the AMRP was determined against the bacterial and fungal strains (Table 3) using the broth microdilution method as described in later section.

**TABLE 3:** Minimum inhibitory concentration (MIC; μg ml^−1^) of *Paenibacillus polymyxa* OSY–EC antimicrobials-rich powder (OSY-EC AMRP) against selected bacterial and fungal strains.

Strain^a^	MIC (µg ml^−1^)
*Escherichia coli* ATCC 8739	156
*E. coli* K12	200
*Enterobacter gergoviae* 565	78
*Klebsiella pneumoniae* 556	120
*Pseudomonas Aeruginosa* ATCC 9027	400
*Serratia marcescens* 562	6,200
*Burkholderia cepacia* ATCC 25416	25,000
*Listeria innocua* ATCC 33090	400
*Staphylococcus aureus* ATCC 6538	400
*Candida albicans* ATCC 10231	1,250
*Aspergillus brasiliensis* ATCC 16404	1,250

a The microbial strains were obtained from the culture collection of the food microbiology laboratory, Department of Food Science and Technology, The Ohio State University, Columbus, Ohio, United States.

### 2.5 Screening Cell-Free Supernatants and Semi-Purified Extracts for Antimicrobial Activity

Antimicrobial bioassay was completed in following media: (i) culture supernatants produced during the initial screening for antimicrobial production in acid whey-based media, and (ii) the semi–purified antimicrobial extract produced during the scaled–up bioreactions (before drying). Aliquots (10 µl) of these media were spotted onto tryptic soy agar (TSA) plates overlaid with soft agar medium (TSA containing 0.75% agar) seeded with an indicator strain (*L. innocua* ATCC 33090, *E. coli* k12, or *C. albicans* P57072). The spotted agar was allowed to absorb the inoculum by leaving the plates undisturbed for 60 min before incubation for 24 h at 37°C for the bacterial indicators or for 48 h at 30°C for *C. albicans* P57072. After incubation, zones of inhibition were observed to determine the antimicrobial activity of spotted aliquots as described in previous studies (He et al., 2007; Campbell et al., 2021). The concentrations of paenibacillin (indicator *L. innocua* ATCC 33090), polymyxin (indicator *E. coli* k12), and fusaricidin (indicator *C. albicans* P57072), in the antimicrobial-containing solutions, were measured in arbitrary units (AU) as follows:

$$Arbitrary\;units\left(AU\right)/ml=\frac{1}{Dilution\;factor\;for\;last\;dilution\;with\;inhibition}x\frac{1000}{volume\;spotted\left(\mu l\right)}$$ (1)

The AU per gram powder was then calculated from the AU/ml of the solution prepared from that powder.

### 2.6 Determining the Minimum Inhibitory Concentration of OSY–EC AMRP

For the MIC determination, the standard CLSI broth microdilution method (Clinical and Laboratory Standards Institute, 2015) was used with modifications. Two–fold serially–diluted AMRP in TSB was prepared and 50 µl aliquots of these preparations were dispensed into the wells of a sterile 96–well plate (Corning, Tewksbury, MA, United States). Overnight cultures of the test organisms (Table 3) were used and their 10^−3^ dilutions in TSB were prepared. Aliquots (50 µl) of diluted cultures were added to the wells of the sterile 96–well plate. The MIC was determined as the lowest concentration of the AMRP which inhibited the visible growth of bacterial or fungal culture after incubation at 37°C for 24 h (for bacteria) or at 22–25°C for 3–6 days (for fungi).

### 2.7 Confirming the Identity of Antimicrobials by Liquid Chromatography

The antimicrobials in supernatants of cultured acid whey and the semi–purified antimicrobial extract were separated using fast protein liquid chromatographic system (Quest 10 plus system; Bio-Rad, Hercules, CA, United States) equipped with a cationic exchange 10 × 100 mm column (ENrich S; Bio-Rad). The elution solvents used were (A) 5 mM phosphate buffer, pH 7 and (B) 5 mM phosphate buffer, pH 7 and 1 M NaCl; these were applied as a gradient, from 0% to 100% solvent B. One ml of active supernatant of cultured acid whey or semi–purified antimicrobial extract was applied to the column after equilibration. Eluted peptides were detected using a multi-UV detector at a 215 nm wavelength (NGC™ Multi-Wavelength Detector; Bio-Rad). Eluted fractions were collected in 1–ml aliquots with a fraction collector (BioFrac™ Fraction; Bio-Rad). Retention times for peaks corresponding to paenibacillin and to a polymyxin–fusaricidin mixture were determined. Fractions with peaks corresponding to paenibacillin and polymyxin–fusaricidin were collected and antimicrobial activities of these agents were confirmed using the bioassay technique described previously (He et al., 2007; Campbell et al., 2021). The indicator strains used were *L. innocua* ATCC 33090, *E. coli* k12, and *C. albicans* P57072 for paenibacillin, polymyxin, and fusaricidin, respectively. The identities of these fractions were confirmed using matrix-assisted laser desorption/ionization time of flight mass spectrometer (MALDI-TOF MS) (UltrafleXtreme; Bruker Daltonics, Billerica, MA, United States) as described previously (Hussein et al., 2020).

### 2.8 Application of OSY–EC AMRP Against *L. innocua* ATCC 33090 and *E. coli* k12 in Different Matrices

#### 2.8.1 Inactivation of the Indicator Strains in a Microbiological Medium

Cells of *L. innocua* ATCC 33090 or *E. coli* k12, grown in TSB for 24 h at 37°C, were harvested by centrifugation, and cell pellets were resuspended in TSB to achieve final population of 10^6^–10^7^ CFU ml^−1^. Cell suspensions were treated with the OSY-EC AMRP at 4x–MIC level (800 and 1,600 μg ml^−1^ for *L. innocua* ATCC 33090 and *E. coli* k12, respectively), and untreated cells (0 μg ml^−1^ OSY–EC AMRP) served as a control. These tests were performed in 96–well microtiter plate (Corning) where each well received 50 µl of AMRP solution to achieve the final concentration, and 50 µl of indicator strain cells. The mixtures were incubated at 37°C for 4 h, and the surviving populations of *L. innocua* and *E. coli* k12, during the incubation, were determined hourly by applying decimal microdilution and plating on TSA.

#### 2.8.2 Inactivation of the Indicator Strains in Pasteurized Milk

Cells of *L. innocua* ATCC 33090 or *E. coli* k12, grown in TSB for 24 h at 37°C, were harvested and cell pellets were resuspended in saline solution (0.85% NaCl). Samples of pasteurized low–fat milk (Horizon, Broomfield, CO, United States) were inoculated with cell suspensions of one of the indicator strains at final population of ∼ 10^5^ CFU ml^−1^ before addition of OSY-EC AMRP at final concentration of 2x–MIC level (400 and 800 µg AMRP ml^−1^ for *L. innocua* ATCC 33090 and *E. coli* k12, respectively). Cells in untreated (0 μg ml^−1^ OSY–EC AMRP) milk served as a control. The control and treated milk samples were incubated at 4°C for 3 days and surviving populations of indicator strains were enumerated daily by plating on TSA.

### 2.9 Elucidation of the Mode of Action of OSY–EC AMRP

For the mechanistic studies, *L. innocua* ATCC 33090 (paenibacillin–sensitive) and *E. coli* k12 (polymyxin–sensitive) were used for studying the antimicrobial mode of action of OSY–EC AMRP produced in the acid whey–based medium. The OSY–EC AMRP contains paenibacillin and polymyxin which are cytoplasmic–membrane active agents. Therefore, experiments to detect leakage of intracellular potassium ions, disruption of outer membrane, and perturbation of inner membrane, were conducted as follows.

#### 2.9.1 Assay for Intracellular Potassium Ion Release

Leakage of intracellular potassium ions from indicator bacteria, which were treated with OSY–EC AMRP solutions, was determined using a fluorescent potassium–sensitive probe (Potassium-binding benzofuran isophthalate, PBFI; Invitrogen, Carlsbad, CA, United States) as described in a previous study (Abdelhamid and Yousef, 2019). Briefly, overnight culture of *L. innocua* ATCC 33090 or *E. coli* k12, grown in TSB, was centrifuged at 8,000 × *g* for 5 min and the cell pellet was resuspended in 5 mM HEPES buffer (Sigma Aldrich) which was supplemented with 5 mM glucose (Fisher Scientific). Portions (50 µl) of the resuspended cells were added to wells of a black 96–well microplate (Corning). This was followed by dispensing the potassium probe to each well at a final concentration of 2 µM. The OSY–EC AMRP was reconstituted in saline solution (0.85% NaCl) and 50 µl of the reconstituted powder was added to each well to achieve final concentrations of 100, 200, 400, 800, 1,600 μg ml^−1^. The OSY–EC AMRP levels ranged from sublethal to lethal levels based on the MIC determined in the current study (Table 3). Untreated cells (0 μg ml^−1^ OSY–EC AMRP) were used as a control. Additional negative control including cells of *L. innocua* or *E. coli* k12 without PBFI or OSY–EC AMRP was used. Release of potassium ions was determined by measurement of fluorescence using a microplate reader (Perkin–Elmer, Wellesley, MA, United States) at excitation and emission wavelength of 346 and 505 nm, respectively. Fluorescence readings were normalized by removing the background fluorescence noise.

#### 2.9.2 Outer Membrane Perturbation Assay

A hydrophobic, uncharged, fluorescent probe (1-*N*-phenylnaphthylamine, NPN; Fisher Scientific) was used to assess the outer membrane permeability as described previously (Elliott et al., 2020) with modifications. The probe, NPN, produces very weak fluorescence in aqueous medium. When the outer membrane is damaged by the act of antimicrobials, NPN reaches the hydrophobic region in the membrane and emits strong fluorescent signals. This criterion is used to evaluate the outer membrane damage in Gram-negative bacteria (Helander and Mattila-Sandholm, 2000). For the assay, overnight cultures of *E. coli* k12 grown in TSB at 37°C, were harvested by centrifugation at 10,000 *g* for 5 min, washed twice with saline solution (0.85% NaCl), and cell pellets were resuspended in saline at a level of 10^7^ CFU ml^−1^. Aliquots (90 µl) from cell suspensions were added into wells of a black 96–well microplate (Corning), and aliquots (10 µl) of NPN at final concentration of 20 µM were added to wells. This was followed by adding 100 µl of OSY–EC AMRP solutions at varying final concentrations (100–1,600 μg ml^−1^) into the wells of the microplate before incubation of the mixture for 90 min at ∼22°C. Untreated cells (0 μg ml^−1^ OSY–EC AMRP) served as the control. Additional negative control including cells of *E. coli* k12 without NPN or OSY–EC AMRP was used. After incubation, fluorescence measurements were performed using a microplate reader (Perkin–Elmer) at excitation/emission wavelength of 340/405 nm. Fluorescence measurements were normalized by subtracting the background noise.

#### 2.9.3 Inner Membrane Perturbation Assay

For investigating inner membrane permeability, a fluorescent DNA–binding and membrane–impermeable dye (Sytox™ Green; Invitrogen) was used as described previously (Elliott et al., 2020) with modifications. The dye produces fluorescence only when entering the cells *via* damaged cytoplasmic membrane and binds to the DNA. For the assay, cell suspensions of *E. coli* k12 and *L. innocua* ATCC 33090, at a final of 10^7^ CFU ml^−1^, were prepared as described in the previous section. Aliquots (99 µl) of cell suspensions were dispended into wells of a black 96–well plate (Corning) before one-µl aliquots of the fluorescent dye, at a final concentration of 2 μM, were added. This was followed by addition of 100-µl aliquots of OSY–EC AMRP solutions, at final concentrations of 100–1,600 μg ml^−1^, to the mixture. Untreated cells (0 μg ml^−1^ OSY–EC AMRP) were used as a control. Additional negative control including cells of *E. coli* k12 without Sytox™ or OSY–EC AMRP was used. The reaction mixtures were incubated for 10 min at 22–25°C before fluorescence was measured using a microplate reader (Perkin–Elmer) at 488 and 530 nm for excitation and emission, respectively. Fluorescence readings were normalized as described in a previous section.

### 2.10 Statistical Analysis

Data were presented as mean ± standard deviation of two biological repeats, and each repeat was performed in triplicate. A statistical software (GraphPad Prism 9.0.0; GraphPad software, San Diego, CA, United States) was used to perform analysis of variance (ANOVA) with Tukey pairwise comparisons to determine significant differences between treatment groups or comparing pairs of treatment. Statistical significance was determined at *p* value <0.05.

## 3 Results

### 3.1 Production of Antimicrobial Peptides in Acid Whey

Unmodified or modified acid whey was used as a growth medium for four bacterial strains known to produce antimicrobial metabolites (Table 2). The unmodified acid whey contained 3.97% lactose, 0.62% protein, 0.5% ash, 0.15% Ca ^2+^ and 0.07% fat; initial pH was 4.6. Using the unmodified acid whey product, growth of the tested antimicrobial-producing bacterial strains was undetectable; hence, no antimicrobial metabolites were detected. Therefore, acid whey neutralization to pH 6–7 was necessary to enable the growth of the antimicrobial producers. With yeast extract supplementation (1% wt/vol) in neutralized acid whey, *B. velezensis* GF610 and *P. polymyxa* OSY–EC strains exhibited considerable activity against the Gram-positive indicator bacterium, *L. innocua* ATCC 33090, after 24 h and 48 h of incubation. *P. polymyxa* OSY–EC was the most capable antimicrobials producer in acid whey, compared to other tested strains (Table 2). *P. polymyxa* OSY–EC produced the highest activity against both the Gram-positive and Gram-negative indicator bacteria. Based on this screening step, optimized condition for production of antimicrobial peptides in acid whey consisted of supplementation with yeast extract (1%), neutralization to pH 7, and incubation for 48 h at 30°C. *P. polymyxa* OSY–EC was selected as the antimicrobial producer strain in an acid whey–based medium for the remainder of the study. Antifungal activity (Table 3) was only identified in acid whey inoculated with OSY–EC strain, but this activity was detectable only after application of the concentration procedures shown in Figure 1.

### 3.2 Scaled–Up Antimicrobials Production and Simultaneous Separation From the Acid Whey–Based Medium

An efficient method that allowed production and separation of antimicrobials simultaneously was developed (Figure 1). In this method, the transition from small scale (20 ml; Table 2) to an 800–ml reaction was achieved. Beads of amberlite-based resin, XAD–7HP, were added to the bioreaction after the first 24 h of incubation to effectively capture the antimicrobials being released by the producer during the remaining 24 h of incubation. Addition of the polymeric beads did not affect the growth of the producer strains nor inhibit the antimicrobial production. The final OSY–EC antimicrobials-rich powder contained 512,000, 32,000 and 8,000 arbitrary unit (AU)/g paenibacillin, polymyxin E, and fusaricidin, as assayed against *L. innocua* ATCC 33090, *E. coli* k12 and *C. albicans* P57072, respectively. Control experiment was completed in which no beads were added during incubation of *P. polymyxa* OSY–EC for 48 h in the acid whey-based medium. In this experiment, bioreaction product was centrifuged and the antimicrobials in the culture supernatant were extracted with the polymeric beads, eluted with acidified ethanol, concentrated under vacuum, and freeze-dried. After antimicrobial bioassay using *L. innocua* ATCC 33090, *E. coli* k12 and *C. albicans* P57072, the resulting powder yielded 10,200, 51,200 and 4,000 AU/g paenibacillin, polymyxin E, and fusaricidin, respectively. The recovered activities, except that for polymyxin E, were considerably smaller than those observed when the beads were added during the last 24 h of incubation.

**FIGURE 2:**
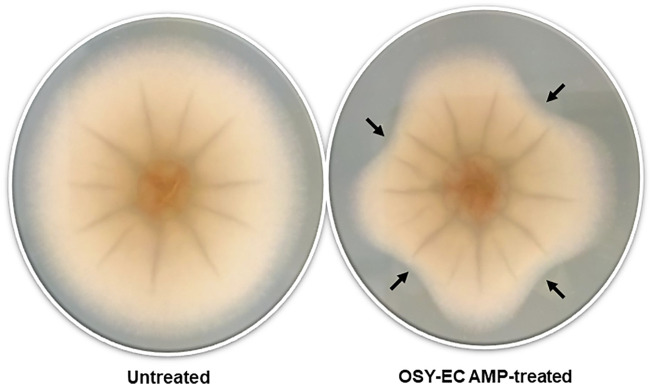
Inhibition of mycelial growth of *Aspergillus brasiliensis* by antimicrobials produced in acid whey-based medium by *Paenibacillus polymyxa* OSY–EC. Representative picture of *A*. *brasiliensis* nonexposed to antimicrobials (Control) or *A*. *brasiliensis* exposed to antimicrobials (Treated) at four different angles indicated by black arrows.

The MIC of the OSY–EC AMRP produced during this large scale bioreaction was determined against selected strains of known problematic bacteria and fungi (Table 3). The OSY–EC AMRP showed potent antimicrobial effect against most of the targeted strains; however, high MIC values were observed in case of *S. marcescens* (6,200 μg ml^−1^) and *B. cepacia* (25,000 μg ml^−1^). It is worth noting that 10–µl aliquots of the resuspended OSY–EC AMRP (50 mg ml^−1^) inhibited the mycelial growth of *A. brasiliensis* on an agar medium (Figure 2). It should be cautioned that the OSY–EC AMRP contain three antimicrobial peptides, which could synergistically inhibit the growth of the tested strains.

### 3.3 Confirming Identities of Antimicrobials by Liquid Chromatography

**FIGURE 3:**
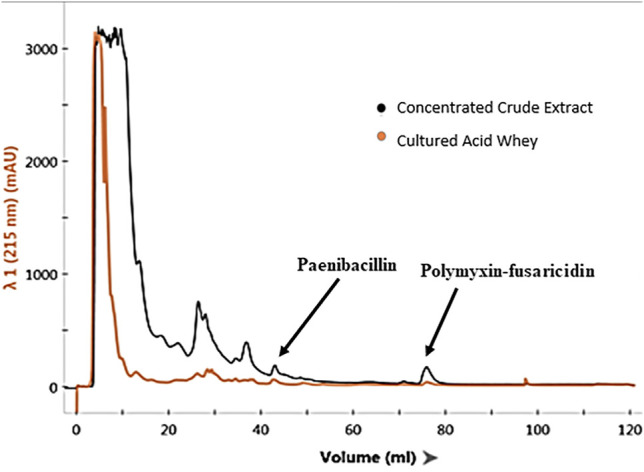
Chromatograms of *Paenibacillus polymyxa* OSY–EC semi–purified antimicrobial extract (black line), compared to cell–free supernatants of cultured acid whey (orange line), after separation on a fast protein liquid chromatography system, equipped with a cationic exchange column and UV detector measuring absorbance at 215 nm. The chromatogram shows the elution profile of paenibacillin and polymyxin–fusaricidin mixture.

**FIGURE 4:**
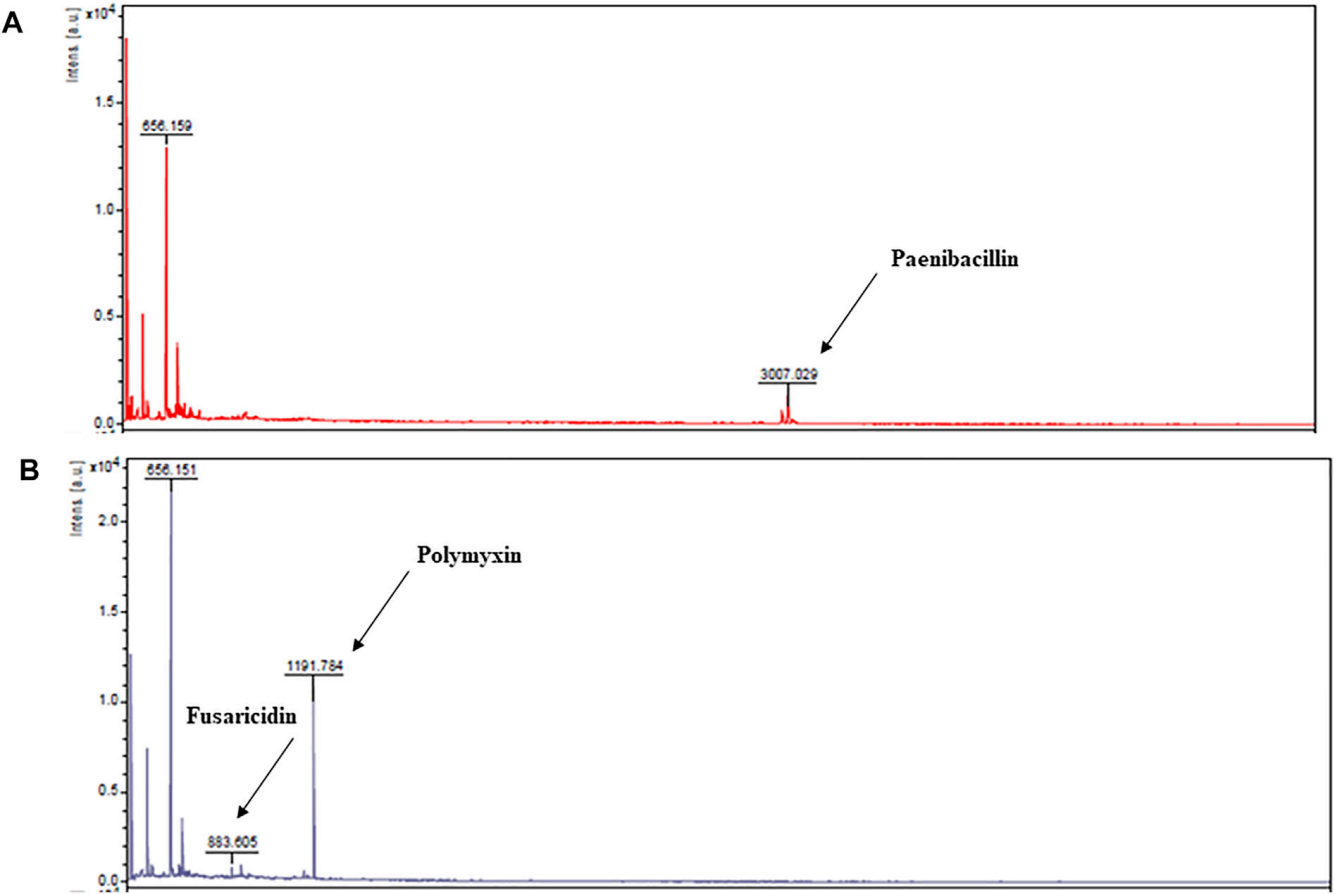
Matrix–assisted laser desorption/ionization time of flight (MALDI-TOF) mass spectra from fast protein liquid chromatography peaks presumed to be paenibacillin **(A)** and polymyxin and fusaricidin **(B)**. The m/z values and assignments are: 3,007.029 [M + Na]^+^, paenibacillin; 883.605 [M + H]^+^, fusaricidin; 1,191.784 [M + Na]^+^, polymyxin E.

Liquid chromatographic analyses were performed to confirm the causes of antimicrobial activities exhibited by the extracts of *P. polymyxa* OSY–EC culture in the modified acid whey. This also helped exclude degradation products of whey components as a source for these antimicrobials. For example, hydrolysis of milk lactoferrin, could lead to the production of lactoferricin, which is an antimicrobial peptide (Gifford et al., 2005). Chromatographic analysis of the antimicrobials produced in modified acid whey by *P. polymyxa* OSY–EC (Figure 3) revealed peaks at known retention times for paenibacillin and for a polymyxin–fusaricidin mixture (Campbell, 2020). The presumed paenibacillin fraction exhibited anti–Gram positive activity (detected by *L. innocua* ATCC 33090) and the polymyxin–fusaricidin fraction produced anti–Gram negative activity (detected by *E. coli* k12) but no antifungal activity was detected. The antifungal activity due to fusaricidin was detected only after concentration of OSY–EC extracts using the polymeric beads. MALDI-TOF MS analysis confirmed the peaks of singly charged ions corresponding to paenibacillin, polymyxin, and fusaricidin, with m/z values of 3,007.029 [M + Na]^+^, 1,191.784 [M + Na]^+^, and 883.605 [M + H]^+^, respectively (Figure 4). In conclusion, three antimicrobials, produced in the modified acid whey media by *P. polymyxa* OSY–EC, were identified and found to contribute to the observed antimicrobial activity of the product.

### 3.4 Inactivation of *L. innocua* and *E. coli* k12 by OSY–EC AMRP in Different Matrices

**FIGURE 5:**
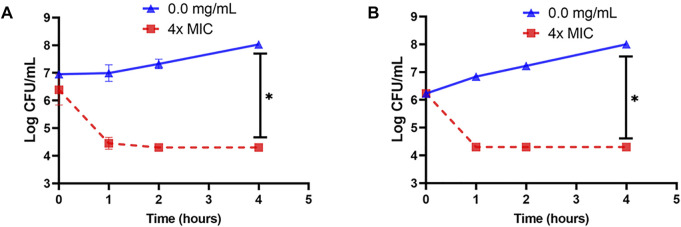
Changes in populations of *Listeria innocua* ATCC 33090 (panel **A**) and *Escherichia coli* k12 (panel **B**) in tryptic soy broth after exposure for up to 4 h at 37°C to 4x–minimum inhibitory concentration (MIC) of the antimicrobials-rich powder (OSY–EC AMRP), prepared from acid whey-based medium inoculated with *Paenibacillus polymyxa* OSY–EC. Data are represented as mean ± standard deviation. Asterisk (*) denotes for significant difference (*p* < 0.05) after 4 h of incubation.

**FIGURE 6:**
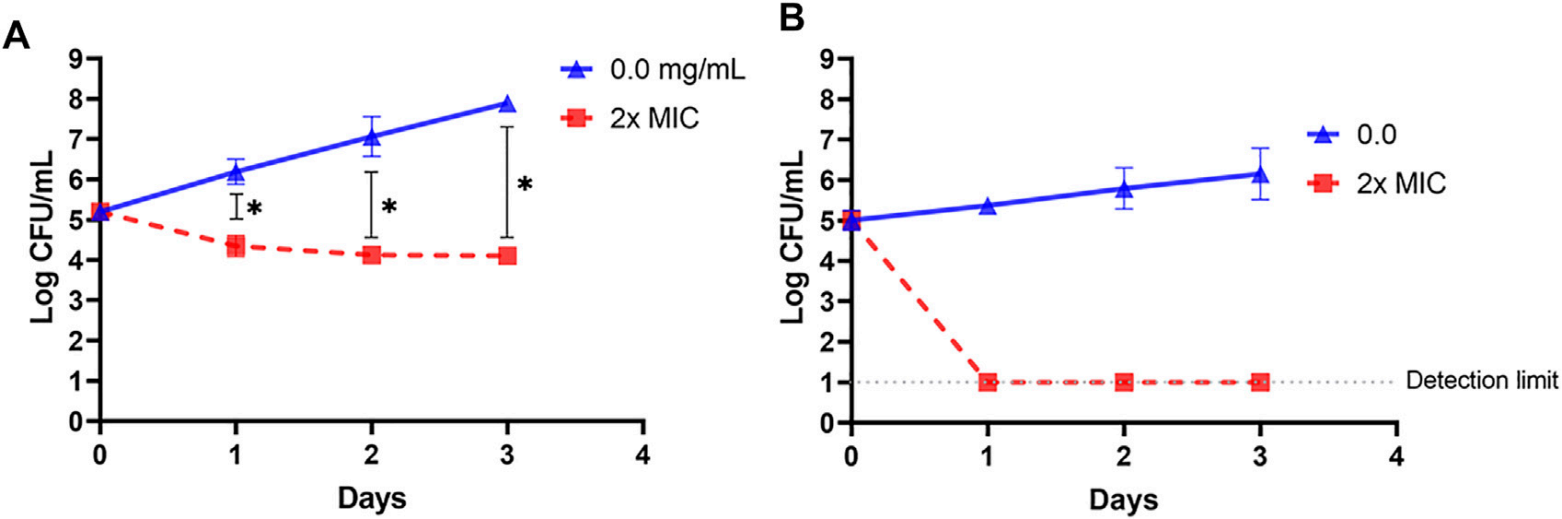
Changes in populations of *Listeria innocua* ATCC 33090 (panel **A**) and *Escherichia coli* k12 (panel **B**) in pasteurized low–fat milk after exposure for up to 3 days at 4°C to 2x–minimum inhibitory concentration (MIC) of the antimicrobials-rich powder (OSY–EC AMRP), prepared from acid whey-based medium inoculated with *Paenibacillus polymyxa* OSY–EC. Data are represented as mean ± standard deviation. Asterisk (*) denotes for significant difference (*p* < 0.05).

Cell suspension of *L. innocua* ATCC 33090 or *E. coli* k12 in TSB was treated with 4x–MIC level of OSY–EC AMRP and the surviving populations during the 4–h incubation at 37°C were presented in Figure 5. Compared to the untreated control, the populations of the two indicators decreased by >2 log CFU ml^−1^ after 1 h of the treatment whereas >4 log CFU ml^−1^ population reduction was observed after 4 h of treatment of the two indicator strains. The successful inactivation of the two indicator bacteria by OSY–EC AMRP in TSB prompted us to explore the inactivation pattern in a food matrix. Milk was selected as a model food considering its susceptibility to contamination and ability to support growth of many pathogenic and spoilage microorganisms. The OSY–EC AMRP at 2x–MIC level was incorporated into sterile milk, inoculated with ∼ 10^5^ CFU ml^−1^ of *L. innocua* or *E. coli* k12, and the inoculated milk was held at 4°C for 3 days. For *L. innocua* ATCC 33090, the population of the bacterium decreased by >2 log CFU ml^−1^ (*p* < 0.05) compared to the untreated control after 1–day incubation (Figure 6A). After 3 days of incubation, *L. innocua* population in the OSY–EC AMRP–treated milk was lower by >3.5 log CFU ml^−1^ (*p* < 0.05), compared to the untreated control. In contrast, *E. coli* k12 population in OSY–EC AMRP–treated milk was below the detection limit (<1 log CFU ml^−1^) after 1 day of incubation and this inactivation level remained until the 3^rd^ day of incubation (Figure 6B). The susceptibility of *E. coli* k12 to OSY–EC AMRP is more profound than that of *L. innocua*. These results demonstrated the potent bactericidal activity of the OSY–EC AMRP against Gram-positive and Gram-negative bacteria in the model food.

### 3.5 The Antimicrobial Mode of Action of OSY–EC AMRP

**FIGURE 7:**
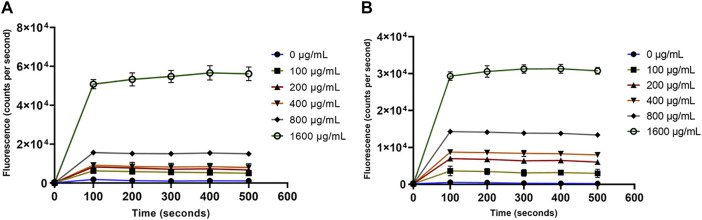
Release of the intracellular potassium ions from *Listeria innocua* ATCC 33090 (panel **A**) and *Escherichia coli* k12 (panel **B**) treated with solutions containing different concentrations (0–1,600 μg/ml) of antimicrobials-rich powder (OSY–EC AMRP), prepared from acid whey-based medium inoculated with *Paenibacillus polymyxa* OSY–EC. The release of intracellular K^+^ was measured as changes in fluorescence at excitation/emission wavelength of 346/505 nm. Data are represented as mean ± standard deviation.

Paenibacillin and polymyxin are the key antimicrobials in OSY–EC AMRP and both are known cytoplasmic membrane–active agents. Demonstrating the action of OSY–EC AMRP against bacterial cytoplasmic membranes further confirms the contribution of paenibacillin and polymyxin to the antimicrobial activity of the powder. Therefore, a panel of membrane perturbation assays was conducted to elucidate the antimicrobial mechanism of action of OSY–EC AMRP, which contained 512,000, or 32,000 AU/g of paenibacillin and polymyxin, respectively, against the indicator strains used in the current study. The MIC values for OSY–EC AMRP were 200 and 400 μg ml^−1^ for *E. coli* k12 and *L. innocua* ATCC 33090, respectively (Table 3). The addition of OSY–EC AMRP at various levels, from sublethal to lethal concentrations, resulted in rapid leakage of intracellular potassium ions from cells of *L. innocua* ATCC 33090 and *E. coli* k12 compared to the untreated control (0 μg ml^−1^ OSY–EC AMRP; *p* < 0.05) (Figure 7). The leakage of potassium ions was a concentration–dependent (*p* < 0.05) and similar trend was observed for the two indicator strains in response to the OSY–EC AMRP treatment.

**FIGURE 8:**
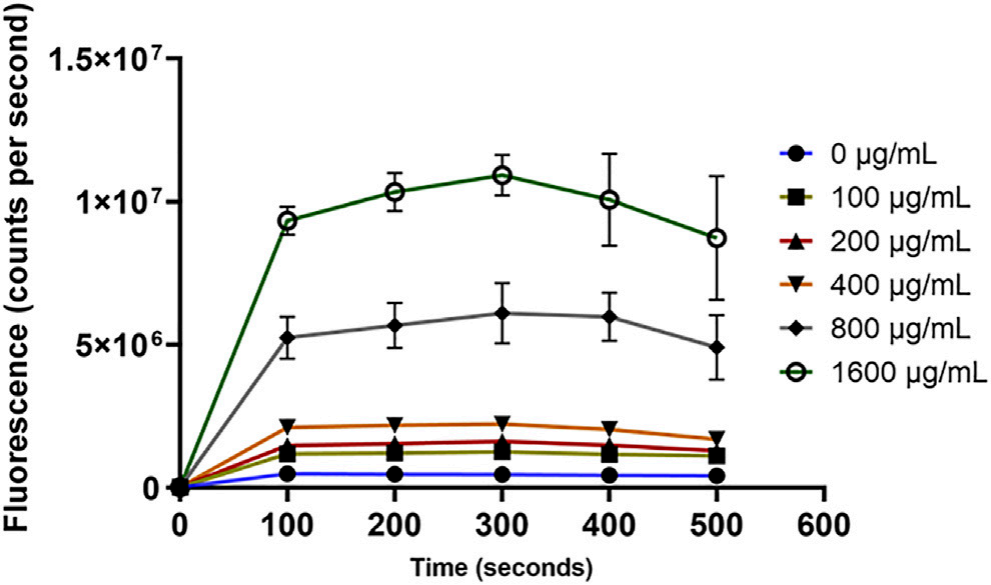
The uptake of 1-*N*-phenylnaphthylamine (NPN) by *Escherichia coli* k12 treated with solutions containing different concentrations (0–1,600 μg/ml) of antimicrobials-rich powder (OSY–EC AMRP), prepared from acid whey-based medium inoculated with *Paenibacillus polymyxa* OSY–EC. The outer membrane disruption in *E. coli* k12 by the peptides was measured as changes in fluorescence at excitation/emission wavelength of 340/405 nm. Data are represented as mean ± standard deviation.

**FIGURE 9:**
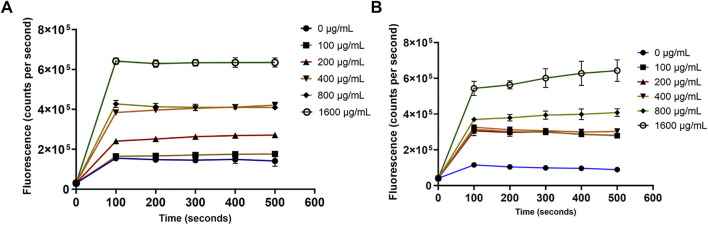
The uptake of Sytox™ green dye by *Listeria innocua* ATCC 33090 (panel **A**) and *Escherichia coli* k12 (panel **B**) treated with solutions containing different concentrations (0–1,600 μg/ml) of antimicrobials-rich powder (OSY–EC AMRP), prepared from acid whey-based medium inoculated with *Paenibacillus polymyxa* OSY–EC. The inner membrane disruption by the peptides was measured as changes in fluorescence at excitation/emission wavelength of 488/530 nm. Data are represented as mean ± standard deviation.

For the outer membrane disruption, it was obvious that the NPN dye produced strong fluorescence signals which significantly (*p* < 0.05) depended on the concentration of OSY–EC AMRP (Figure 8). This increased fluorescence is attributed to the uptake of NPN by the disrupted outer membrane of *E. coli* k12 (Figure 8). Similarly, the cytoplasmic membrane permeability was compromised by the OSY–EC AMRP (Figure 9); this was evident by the fluorescence signals of Sytox™ Green dye which increased proportionally (*p* < 0.05) to the concentration of OSY–EC AMRP added to the cell suspension of the indicator strains. Thus, cell membranes of the two indicator strains were apparently disrupted by the OSY–EC AMRP. Collectively, these results proved that OSY–EC AMRP caused damages to membranes of targeted cells owing to the membrane–active peptides, paenibacillin (Anti–*L. innocua*) and polymyxin E (anti–*E. coli*).

## 4 Discussion

Use of acid whey to produce antimicrobials by beneficial bacteria may be considered an innovative approach for valorization of this dairy waste. Although dairy processors developed many applications for cheese sweet whey, they have shown less interest in acid whey due to its different characteristics. Industrial applications of sweet whey include its bioconversion into value–added products such as organic acids (Pandey et al., 2019; Ricciardi et al., 2019), aroma compounds (Nadal et al., 2009), biopolymers (Lule et al., 2016; Lappa et al., 2019), bio–alcohols (Becerra et al., 2015; Pescuma et al., 2015), and biogas (Pescuma et al., 2015; Pagliano et al., 2019). The microbial species involved in these bioconversions varied depending on the target product. Microbial species used in cheese whey valorization include *Saccharomyces cerevisiae*, *Clostridium* spp., *Leuconostoc mesenteroides*, *Xanthomonas campestris*, and some lactobacilli such as *Lactobacillus reuteri*, *L. sakei*, and *L. plantarum* (Zotta et al., 2020). Despite this mounting research, less attention has been given to the utilization of acid whey. In the current work, acid whey has been transformed into valuable antimicrobial powder which may be used industrially in preservation of food or healthcare products, or in antimicrobial packaging applications.

In the current study, acid whey without pH neutralization did not support the growth of *B. velezensis* strains or *P. polymyxa* OSY–EC because these bacteria require pH 6, or higher, to grow efficiently (Li et al., 2018; Patowary and Deka, 2020); this is a contrary behavior to that of lactic acid bacteria, which endure and grow in acidic environment (Hutkins and Nannen, 1993). Neutralization and yeast extract supplementation of acid whey improved considerably the growth of the test bacteriocinogenic bacteria and their ability to produce antimicrobials in the modified medium. Yeast extract is known to contain growth factors and free amino acids such as glutamic acid, aspartic acid, alanine, leucine, lysine, valine, and arginine, which act as stimulants for growth and precursors for antimicrobial peptides production (Abbasiliasi et al., 2017). Acid whey–based medium allowed the production of broad–spectrum antimicrobials by *P. polymyxa* OSY–EC. This can be attributed, in part, to the fact that *P. polymyxa* OSY–EC was developed as a beneficial mutant from the parent strain, *P. polymyxa* OSY–DF, which originally produced paenibacillin at low levels (Campbell et al., 2021). In that study, *P. polymyxa* OSY–EC exhibited enhanced paenibacillin titer and maintained stability of paenibacillin production after several passages (Campbell et al., 2021). Thus, the genetic and phenotypic stability of the OSY–EC strain is advantageous for acid whey utilization and antimicrobial peptide production.

A method that facilitated capture of antimicrobial peptides during growth of *P. polymyxa* OSY–EC in acid whey–based medium was successfully developed in the present work (Figure 1). The beads of the polymeric–resin, XAD–7HP offered a good capture affinity for the antimicrobials from the cultured acid whey during the bioreactions. This was evident when the incubated acid whey–beads mixture was filtered, and the filtrate was tested for the presence of antimicrobials; the filtrate showed no antimicrobial activity (data not shown) whereas solvent extracts of the beads exhibited potent antimicrobial activity. This single–step process for production and concentration of antimicrobials yielded 512,000, 32,000 and 8,000 AU/g paenibacillin, polymyxin E, and fusaricidin, respectively. Compared to the antimicrobial amounts produced by the two–step process (i.e., bioreaction step, followed by the step of separation by polymeric beads), the single–step process increased fusaricidin and paenibacillin yields by 2- and 50-times, respectively. The single–step process for production and concentration of antimicrobials provides benefits in the context of industrial applications due to its simplicity, quickness, and ease of recovery of antimicrobials.

The MIC results of OSY–EC AMRP against the tested microbial strains is relatively high, compared to the standard MIC breakpoints used in antimicrobial testing (Clinical and laboratory standards institute, 2022). However, the AMRP could act as an antimicrobial–rich source for further concentration and purification of the produced antimicrobial peptides; this could lower the MIC levels of the product considerably.

Separation and purification of individual peptides in the AMRP requires the application of liquid chromatographic procedures which are resource-intensive processes. Peptides of microbial origin are often produced in small quantities (Schreiber et al., 2017), thus low yield is expected after these downstream processes. Alternative methods have been proposed for large scale production of antimicrobial peptides; these include solid–phase peptide synthesis (SPPS) *via* chemical means (Bédard and Biron, 2018). Even though SPPS is viable for small and medium sized peptides (5–50 residue), only few bacteriocins were successfully prepared at satisfactory levels using the chemical method. Chemical synthesis of microbially-produced antimicrobial peptides may by hampered by product loss of bioactivity and complexity of the resulting peptide structure (Bédard and Biron, 2018). Hence, new chemical–based approaches which overcome these challenges can provide solution for efficient preparation of the bioactive peptides in large quantities.

The application of OSY–EC AMRP in different matrices (TSB and milk) showed potent antagonistic activity against *L. innocua* and *E. coli* K12. In a previous study, Sakacin-A was produced in cheese whey and the researchers applied Sakacin-A–coated paper for packaging of soft cheese inoculated with *L. innocua* (Rollini et al., 2020). In their study, the researchers found that Sakacin-A caused 1–log reduction in *L. innocua* population after 21 days of incubation. Comparably, the current work demonstrated that the populations of the indicator strains decreased by >3.5 log CFU ml^−1^ in milk after 3 days of treatment (Figure 6). Thus, the OSY–EC antimicrobials-rich powder produced in the acid whey–based medium could be a compelling antimicrobial preparation for further biopreservation applications. The fact that OSY–EC antimicrobial powder contains three active peptides is advantageous for biopreservation, particularly if these peptides can act synergistically against targeted microorganisms. In an investigation, fusaricidin A was found active against both fungi and Gram-positive bacteria (Kajimura and Kaneda, 1996), whereas the combination of polymyxin with nisin, a lantibiotic similar to paenibacillin, was synergistic against *Pseudomonas aeruginosa* biofilms (Field et al., 2016). In addition to its antibacterial activity, polymyxin was reported to have antifungal activity against species of *Candida*, *Aspergillus*, *Fusarium*, and other molds (Yousfi et al., 2019).

Paenibacillin and polymyxin E are the principal antimicrobial components of OSY–EC AMRP. The current work indicated the ability of the two peptides to disrupt cell membranes of the indicator strains (Figures 7–9). This is consistent with previous findings that polymyxin E disrupts bacterial cell membrane *via* targeting the lipopolysaccharides (Ayoub Moubareck, 2020; Sabnis et al., 2021) whereas paenibacillin M152–P4, a new compound produced by a *Paenibacillus* spp. strain, was found to cause membrane permeabilization against *Enterococcus faecium* ATCC 51559 (Jangra et al., 2019). Paenibacillin and polymyxin E are cationic peptides that have amphiphilic nature (He et al., 2007; Velkov et al., 2010), which contribute to their antimicrobial activity by binding to the anionic surfaces of Gram-positive and Gram-negative bacteria, respectively. This antimicrobial–bacterial surface interaction disrupts membranes permeabilities and induce leakage of intracellular constituents (e.g., K^+^) leading to bacterial cell death.

## 5 Conclusion

Cheese acid whey is a dairy industry waste product that has high–disposal cost and undesirable environmental impact. The current study provided a straightforward process for utilizing acid whey to produce broad–spectrum antimicrobial peptides using the versatile beneficial bacterium, *P. polymyxa* OSY–EC. This microbial conversion is a promising strategy to avail potent antimicrobials for food preservation while valorizing acid whey. Future studies should be performed to improve the concentration and purification of antimicrobials from spent acid whey and assess their utility in various food products.

## Data Availability

The original contributions presented in the study are included in the article, further inquiries can be directed to the corresponding author.
